# Crystal structures of metallocene complexes with uranium–germanium bonds

**DOI:** 10.1107/S2056989021011269

**Published:** 2021-11-09

**Authors:** Michael L. Tarlton, Steven P. Kelley, Justin R. Walensky

**Affiliations:** a Department of Chemistry, University of Missouri-Columbia, Columbia, MO 65211, USA

**Keywords:** crystal structure, actinide, germ­anium, main group, organometallic chemistry

## Abstract

The first structural examples of complexes with uranium–germanium bonds are presented, namely, bis­[3,5-bis­(tri­fluoro­meth­yl)phenyl-2κ*C* ^1^](hydrido-2κ*H*)(iodido-1κ*I*)bis­[1,1(η^5^)-penta­methyl­cyclo­penta­dien­yl]germaniumuranium(*Ge*—*U*), [GeU(C_10_H_15_)_2_(C_8_H_3_F_6_)_2_HI], and bis­[3,5-bis­(tri­fluoro­meth­yl)phenyl-2κ*C* ^1^](fluorido-1κ*I*)(hydrido-2κ*H*)bis­[1,1(η^5^)-penta­methyl­cyclo­penta­dien­yl]germaniumuranium(*Ge*—*U*), [GeU(C_10_H_15_)_2_(C_8_H_3_F_6_)_2_FH]. The two complexes both have a long U—Ge bond [distances of 3.0428 (7) and 3.0524 (7) Å].

## Abstract 2

The first structural examples of complexes with uranium–germanium bonds are presented. The two complexes both have a long U—Ge bond [distances of 3.0428 (7) and 3.0523 (7) Å.

## Chemical context

While actinide complexes with heavier main-group elements have been studied with the chalcogen and pnictogen groups, the tetrel series has not been examined in nearly as much detail. Actinide–heavier main-group element bonds have been of inter­est to our group and others, for three primary reasons. First is the exploration of the energy-driven-covalency concept, which shows increased covalent-bonding character going down a group (Walensky *et al.*, 2010 ▸; Neidig *et al.*, 2013 ▸; Su *et al.*, 2018 ▸). Second, despite increased covalency, bond strength does not scale with covalency, hence the weaker, more polarized bonds with heavier main-group elements should afford greater reactivity (Kaltsoyannis, 2013 ▸). Finally, the fundamental chemistry obtained by the structure, bonding, and reactivity of these understudied metals advances our knowledge of the periodic table and helps to elucidate new and exciting properties.

The coordination chemistry of *f* elements with heavier tetrel elements (Si, Ge, Sn, Pb) is quite rare (Réant *et al.*, 2020*b* ▸), and their reactivity is virtually unknown. With respect to the actinides, there are two reports of actin­ide–silicon bonds without structural data (King & Marks, 1995 ▸; Radu *et al.*, 1995 ▸), and two structurally characterized uranium–silicon bonds with anionic silyl ligands (Diaconescu *et al.*, 2001 ▸; Réant *et al.*, 2020*a* ▸) and two more with neutral silylene ligands (Brackbill *et al.*, 2020 ▸). In the 1990s, the reaction of (C_5_H_5_)_3_UCl with K*E*Ph_3_, *E* = Si, Ge, Sn, was conducted by Porchia and co-workers to form uranium–tetrel bonds, and their reactivity with isocyanides was described (Porchia *et al.*, 1986 ▸, 1987 ▸, 1989 ▸). Finally, the Boncella group has more recently reported a second uranium–tin bond (Winston *et al.*, 2016 ▸). We have found that protonolysis reactions with primary pnictines have resulted in the formation of actinide–pnictido bonds (Behrle & Walensky, 2016 ▸; Vilanova *et al.*, 2017 ▸; Tarlton *et al.*, 2020 ▸, 2021 ▸), so we decided to utilize a secondary germane in the same strategy. However, the issue is the protonic *versus* hydridic nature of the *E*—H bonds, and hence we used 3,5-(CF_3_)_2_C_6_H_3_-substituted germane to obtain a more protonic hydrogen. Herein, we report the structural characterization of uranium–germanium bonds with a secondary germanido ligand. When attempting to form the germylene, a C—F bond-activated product is obtained, indicating the reactive nature of these weak uranium–germanium bonds. 
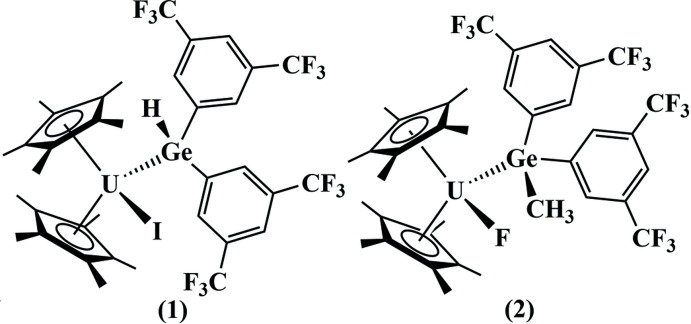


## Structural commentary

**Figure 1:**
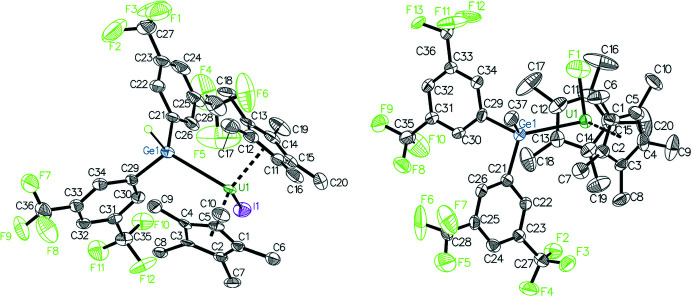
50% probability ellipsoid plots of compounds **1** (*left*) and **2** (*right*). The Ge—H hydrogen atom in **1** is shown as a green circle, all other H atoms and minor disordered parts are omitted for clarity. Elements are color coded as follows: C = black, F = yellow–green, Ge = dark blue, I = purple, U = dark green.

The solid-state structure of each complex was definitively determined by X-ray crystallography to elucidate the first uranium–germanium bond (Fig. 1 ▸). Both structures have similar geometries in which the U atom is coordinated to two η^5^-coordinating Cp* ligands, a halide ligand, and the germanido ligand, which coordinates only through the germanium atom in both cases. Geometric parameters involving the U—Ge distances and Ge-centered angles are given in Table 1 ▸. Each U—Ge bond is within the sum of the covalent radii of 3.16 Å (Cordero *et al.*, 2008 ▸). Both complexes are distorted tetra­hedra with the Cp* ligands occupying single vertices. The angles between the Cp* mean planes are similar in both structures [133.4 (3)° for **1** and 137.8 (3)° for **2**], which is significantly larger than the ideal tetra­hedral angle as expected for two adjacent, sterically bulky ligands. The uranium-centered angles between the halide and Ge atoms in both structures are consequently distorted to smaller angles [88.06 (2)° for **1** and 88.92 (14)° for **2**]. The 3,5-(CF_3_)_2_C_6_H_3_ rings are oriented significantly differently in the two structures. In **1** the rings are roughly consistent with a mirror plane passing through the U, Ge, and I atoms, and their mean planes inter­sect at an angle of 72.8 (2)°. In **2** they have an unsymmetrical orientation, which appears to be the result of rotation of the germanido ligand to reduce repulsion between the Cp* and Ge—CH_3_ groups, and the mean planes of the rings inter­sect at an angle of 66.1 (1)°.

## Supra­molecular features

**Figure 2:**
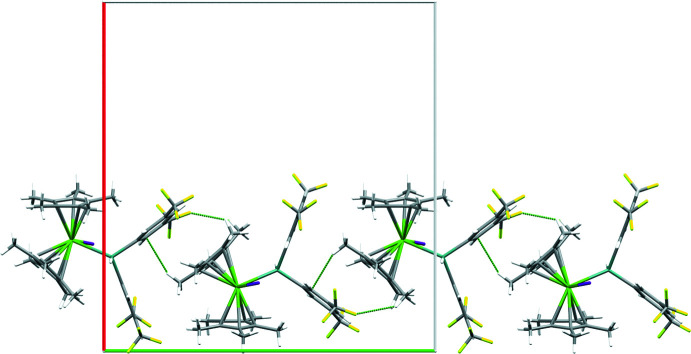
Packing plot viewed down *c* showing complementary inter­actions between 3,5-(CF_3_)_2_C_6_H_3_ and Cp* rings in **1**. Dashed green lines indicate short (less than the sum of the van der Waals radii) contacts. Elements color coded as in Fig. 1 ▸. Axes color coded as follows: *a* = red, *b* = green.

Compound **1** crystallizes in the space group *C*2/*c* with *Z′* = 1. Each mol­ecule makes short (less than the sum of the van der Waals radii) contacts to six neighboring mol­ecules. Two of these neighbors inter­act through donating or receiving a weak Ge—H⋯I hydrogen bond (Table 2 ▸), which forms the basis of an infinite chain parallel to *c*. The two 3,5-(CF_3_)_2_C_6_H_3_ rings bonded to the Ge atom form a cavity, which complements the shape of the two Cp* groups, resulting in two neighboring mol­ecules encapsulating or residing within this cavity and forming chains parallel to the *b*-axis direction (Fig. 2 ▸). The layers formed by these two inter­actions stack along the *c*-axis direction with adjacent layers making contact through like–like inter­actions between –CF_3_ or Cp* groups, which are likely only weakly attractive or repulsive in nature. The phenyl rings are unsymmetrical in their inter­actions with one making a larger number of short contacts; the ring which makes fewer contacts has rotational disorder of both –CF_3_ groups, which could be modeled over two positions in one case [modeled at occupancies of 0.536 (8) and 0.464 (8)] and is indicated by the shape of the displacement ellipsoids in the other case.

**Figure 3:**
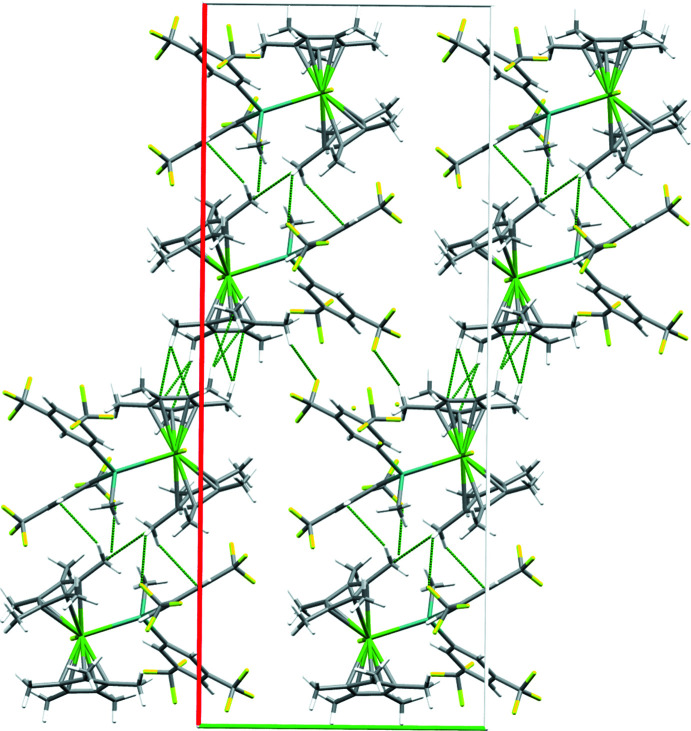
Packing plot showing formation of close-packed layers in **2**. Dashed green lines indicate short contacts. Elements color coded as in Fig. 1 ▸; unit-cell axes color coded as in Fig. 2 ▸.

Compound **2** crystallizes in the monoclinic space group *C*2/*c* with *Z*′ = 1. Each mol­ecule makes short contacts to seven neighboring mol­ecules. One neighboring mol­ecule inter­acts to form a centrosymmetric dimer through ion–dipole inter­actions between Cp* –CH_3_ and aromatic C atoms. A second neighboring mol­ecule also inter­acts across an inversion center through similar inter­actions between the other Cp* ligand and one of the phenyl rings. The remaining mol­ecules only inter­act through C—H⋯F contacts (Table 2 ▸). The inter­actions involving the Cp* ligands appear to be the strongest and organize the mol­ecules into tightly packed layers which are parallel to the (001) family of planes (Fig. 3 ▸) , and these layers are bridged through the C—H⋯F inter­actions into a three-dimensional network. As with **1**, one of the 3,5-(CF_3_)_2_C_6_H_3_ rings does not participate as strongly in inter­molecular inter­actions and has disordered –CF_3_ groups, one of which could be modeled over two positions (occupancies 0.75 and 0.25).

## Database survey

The uranium–germanium bond distance in **1** of 3.0427 (8) Å is similar to the 3.0688 (8) and 3.091 (3) Å uranium–silicon bonds in [(C_5_H_4_SiMe_3_)_3_U{Si(SiMe_3_)_3_}] (Réant *et al.*, 2020*a* ▸, CSD refcode: CUTZUP) and [U{N(^*t*^Bu)C_6_H_3_-3,5-Me_2_}_3_{Si(SiMe_3_)_3_}] (Diaconescu *et al.*, 2001 ▸, CSD refcode: XOKQET), respectively. The 2.9512 (7) Å uranium–iodide bond length is nearly identical to the value of 2.9868 (9) in [(C_5_Me_5_)_2_UI_2_] (Graves *et al.*, 2008 ▸, CSD refcode: ROJNOU) and 2.953 (2) Å in [{C_5_H_3_(SiMe_3_)_2_}_2_UI_2_] (Blake *et al.*, 1995 ▸, CSD refcode: ZEYZIM). In **2**, the U—Ge bond distance is 3.0523 (7) Å with a U—F distance of 2.242 (5) Å. The U—F bond distance is quite long compared to the 2.06 (1)-2.15 (1) Å previously observed in U^IV^ metallocenes (Thomson *et al.*, 2010 ▸, CSD refcode: TABJAJ; Kagan *et al.*, 2018 ▸, CSD refcodes: SEYKEP, SEYKIT, SEYKOZ, SEYKUF; Boreen *et al.*, 2020 ▸, CSD refcodes: BUQMAE, BUQMEI), but shorter than the sterically crowded complex, (C_5_Me_5_)_3_UF, which has a U—F bond length of 2.43 (2) Å (Evans *et al.*, 2000 ▸, CSD refcode: XENQUC).

## Synthesis and crystallization

**Figure 4:**
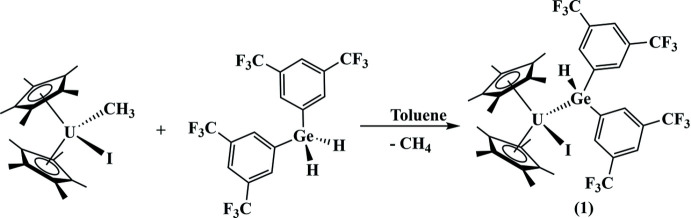
Synthesis of compound **1**.

The reaction of (C_5_Me_5_)_2_U(I)(CH_3_) (Rungthanaphatsophon *et al.*, 2018 ▸) with one equivalent of H_2_ *E*[3,5-(CF_3_)_2_C_6_H_3_)_2_] (Bender IV *et al.*, 1997 ▸) in toluene at room temperature produces a dark-red solution (Fig. 4 ▸). The solution was allowed to stir for 4 h after which the solvent was removed, and the product recrystallized from a saturated toluene solution at 248 K.

**Figure 5:**
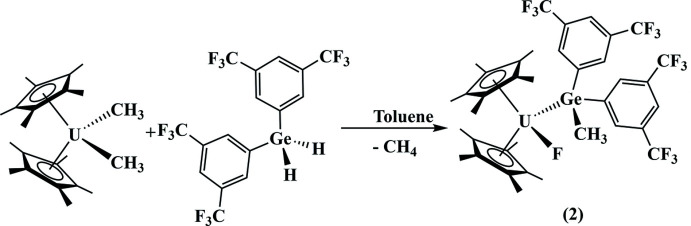
Synthesis of compound **2**.

The reaction of (C_5_Me_5_)_2_U(CH_3_)_2_ with one equivalent of H_2_ *E*[3,5-(CF_3_)_2_C_6_H_3_)_2_] (Bender IV *et al.*, 1997 ▸) in toluene at 333 K produces a dark-red solution (Fig. 5 ▸). The solution was allowed to stir overnight after which the solvent was removed, and the product recrystallized from a saturated toluene solution at 248 K. No byproducts could be found that led us to a plausible mechanism of C—F bond activation.

## Refinement

The crystal structure of **1** was solved by an iterative dual space approach as implemented in *SHELXT*. All full occupancy non-hydrogen atoms could be located from the difference map refined anisotropically. In one of the disordered –CF_3_ groups, two sets of fluorine atoms could be located from the difference map. The other –CF_3_ group on the same mol­ecule has prolate ellipsoids, which indicates disorder of this group as well, but attempts to model additional F-atom positions using chemically reasonable difference map peaks resulted in extremely poor geometries and displacement parameters. All C—F distances for this mol­ecule were restrained to 1.33 (1) Å, and the intra­molecular F⋯F distances were restrained to be equal within ± 0.01 Å. For the –CF_3_ group modeled over two positions, the three pairs of related F atoms each had their anisotropic displacement parameters constrained to be equal. The occupancies of the major and minor parts refined to 53.6 and 46.4% (± 0.8%) and were constrained to sum to 100%. The H atom bonded to Ge was located from the difference map, its coordinates were allowed to refine, and its isotropic displacement parameter was constrained to ride on the carrier atom. The structure also contained large residual difference map peaks in chemically non-reasonable positions. Some of these peaks occur at distances from the U atom very close to the U—I bond and have *x* or *y* coordinates equal to the I atom. Given the layer packing of this structure, these peaks most likely correspond to packing defects where layers are occasionally shifted relative to each other resulting in superposition of mol­ecules related by rotation or reflection. These peaks could not be modeled, but truncating the data to a resolution of 0.77 Å greatly reduced their intensity.

The crystal structure of **2** was solved by an iterative dual space method as implemented in *SHELXT*. All non-hydrogen atoms were located from the difference map and refined anisotropically. For the disordered –CF_3_ group both sets of F atoms were located from the difference map. The occupancies were manually adjusted until the isotropic thermal parameters were approximately equal, which occurred at 75% for the major part and 25% for the minor part. The major part could be refined anisotropically without restraints; the minor part failed to converge in an anisotropic refinement and was left isotropic.

All other refinement details and software are summarized in Table 3 ▸.

## Supplementary Material

Crystal structure: contains datablock(s) 1, 2. DOI: 10.1107/S2056989021011269/vm2256sup1.cif

Structure factors: contains datablock(s) 1. DOI: 10.1107/S2056989021011269/vm22561sup2.hkl

Structure factors: contains datablock(s) 2. DOI: 10.1107/S2056989021011269/vm22562sup3.hkl

CCDC references: 2117996, 2117995

Additional supporting information:  crystallographic information; 3D view; checkCIF report

## Figures and Tables

**Table 1:** Selected geometric parameters (Å, °) for **1** and **2**

Parameter	**1**	**2**
U1—Ge1	3.0428 (7)	3.0524 (7)
U1—Ge1—C21	118.5 (2)	116.65 (17)
U1—Ge1—C29	116.73 (18)	117.87 (16)

**Table 2:** Selected geometric parameters (Å, °) for **1** and **2**

Contact	Distance (*D*⋯*A*)	Distance (*D*—H⋯*A*)
**1**		
Ge1—H⋯I1	4.5876 (9)	3.16 (8)
C7—H⋯F12	3.314 (9)	2.524
C16—H⋯C30	3.70 (1)	2.79
C20*B*—H⋯F7	3.44 (1)	2.65
**2**		
C7—H⋯F3	3.403 (8)	2.652
C7—H⋯C24	3.536 (9)	2.863
C7⋯C7	3.36 (1)	
C10—H⋯F3	3.216 (8)	2.617
C16—H⋯C19	3.84 (1)	2.87
C18—H⋯F10	3.446 (8)	2.543
C20—H⋯C11	3.57 (1)	2.80
C20—H⋯C12	3.654 (9)	2.752

H⋯*A* distances involving riding H atoms are rounded to the precision of the *D*⋯*A* distance.

**Table 3:** Experimental details

	**1**	**2**
Crystal data		
Chemical formula	[GeU(C_10_H_15_)_2_(C_8_H_3_F_6_)_2_HI]	[GeU(C_10_H_15_)_2_(C_8_H_3_F_6_)_2_FH]
*M*_r_	1135.17	1041.30
Crystal system, space group	Monoclinic, *C*2/*c*	Monoclinic, *C*2/*c*
Temperature (K)	100	100
*a*, *b*, *c* (Å)	23.647 (2), 21.123 (2), 16.8132 (18)	34.160 (3), 13.5237 (11), 16.2986 (13)
β (°)	109.727 (3)	95.028 (2)
*V* (Å^3^)	7905.1 (14)	7500.4 (10)
*Z*	8	8
Radiation type	Mo *K*α	Mo *K*α
μ (mm^−1^)	5.71	5.21
Crystal size (mm)	0.64 × 0.63 × 0.53	0.10 × 0.10 × 0.08

Data collection		
Diffractometer	Bruker VENTURE CMOS area detector	Bruker APEXII CCD
Absorption correction	Multi-scan (*AXScale*; Bruker, 2015 ▸	Multi-scan (*SADABS*; Bruker, 2015 ▸)
*T*_min_, *T*_max_	0.136, 0.563	0.501, 0.562
No. of measured, independent and observed [*I* > 2σ(*I*)] reflections	103146, 9105, 7700	65643, 7720, 5563
*R*_int_	0.076	0.081
(sin θ/λ)_max_ (Å^−1^)	0.651	0.627

Refinement		
*R*[*F* ^2^ > 2σ(*F* ^2^)], *wR*(*F* ^2^), *S*	0.045, 0.112, 1.04	0.041, 0.094, 1.05
No. of reflections	9105	7720
No. of parameters	483	492
No. of restraints	24	0
H-atom treatment	H atoms treated by a mixture of independent and constrained refinement	H-atom parameters constrained
Δρ_max_, Δρ_min_ (e Å^−3^)	2.63, −2.00	1.68, −0.78

Computer programs: *APEX3* and *SAINT* Bruker, 2015 ▸), *SHELXT* (Sheldrick, 2015*a* ▸), *SHELXL* (Sheldrick, 2015*b* ▸), and *OLEX2* (Dolomanov *et al.*, 2009 ▸).

## supplementary crystallographic information

### Bis[3,5-bis(trifluoromethyl)phenyl-2κC1](hydrido-2κH)(iodido-1κI)bis[1,1(η5)-pentamethylcyclopentadienyl]germaniumuranium(Ge—U) (1) Crystal data

**Table:**

[GeU(C_10_H_15_)_2_(C_8_H_3_F_6_)_2_HI]	*F*(000) = 4304
*M**_r_* = 1135.17	*D*_x_ = 1.908 Mg m^−^^3^
Monoclinic, *C*2/*c*	Mo *K*α radiation, λ = 0.71073 Å
*a* = 23.647 (2) Å	Cell parameters from 9630 reflections
*b* = 21.123 (2) Å	θ = 2.3–27.5°
*c* = 16.8132 (18) Å	µ = 5.71 mm^−^^1^
β = 109.727 (3)°	*T* = 100 K
*V* = 7905.1 (14) Å^3^	Block, clear dark red
*Z* = 8	0.64 × 0.63 × 0.53 mm

### Bis[3,5-bis(trifluoromethyl)phenyl-2κC1](hydrido-2κH)(iodido-1κI)bis[1,1(η5)-pentamethylcyclopentadienyl]germaniumuranium(Ge—U) (1) Data collection

**Table:**

Bruker VENTURE CMOS area detector diffractometer	9105 independent reflections
Radiation source: Incoatec IMuS microfocus Mo tube	7700 reflections with *I* > 2σ(*I*)
Mirror optics monochromator	*R*_int_ = 0.076
shutterless ω and phi scans	θ_max_ = 27.6°, θ_min_ = 2.1°
Absorption correction: multi-scan (*AXScale*; Bruker, 2015	*h* = −30→30
*T*_min_ = 0.136, *T*_max_ = 0.563	*k* = −27→27
103146 measured reflections	*l* = −21→21

### Bis[3,5-bis(trifluoromethyl)phenyl-2κC1](hydrido-2κH)(iodido-1κI)bis[1,1(η5)-pentamethylcyclopentadienyl]germaniumuranium(Ge—U) (1) Refinement

**Table:**

Refinement on *F*^2^	Primary atom site location: dual
Least-squares matrix: full	Secondary atom site location: difference Fourier map
*R*[*F*^2^ > 2σ(*F*^2^)] = 0.045	Hydrogen site location: mixed
*wR*(*F*^2^) = 0.112	H atoms treated by a mixture of independent and constrained refinement
*S* = 1.03	*w* = 1/[σ^2^(*F*_o_^2^) + (0.0386*P*)^2^ + 173.3198*P*] where *P* = (*F*_o_^2^ + 2*F*_c_^2^)/3
9105 reflections	(Δ/σ)_max_ = 0.001
483 parameters	Δρ_max_ = 2.63 e Å^−^^3^
24 restraints	Δρ_min_ = −2.00 e Å^−^^3^

### Bis[3,5-bis(trifluoromethyl)phenyl-2κC1](hydrido-2κH)(iodido-1κI)bis[1,1(η5)-pentamethylcyclopentadienyl]germaniumuranium(Ge—U) (1) Special details

**Table:**

Geometry. All esds (except the esd in the dihedral angle between two l.s. planes) are estimated using the full covariance matrix. The cell esds are taken into account individually in the estimation of esds in distances, angles and torsion angles; correlations between esds in cell parameters are only used when they are defined by crystal symmetry. An approximate (isotropic) treatment of cell esds is used for estimating esds involving l.s. planes.

### Bis[3,5-bis(trifluoromethyl)phenyl-2κC1](hydrido-2κH)(iodido-1κI)bis[1,1(η5)-pentamethylcyclopentadienyl]germaniumuranium(Ge—U) (1) Fractional atomic coordinates and isotropic or equivalent isotropic displacement parameters (Å^2^)

**Table:**

	*x*	*y*	*z*	*U*_iso_*/*U*_eq_	Occ. (<1)
U1	0.18303 (2)	0.40289 (2)	0.22613 (2)	0.02210 (7)	
I1	0.19437 (2)	0.46585 (3)	0.07554 (3)	0.04105 (13)	
Ge1	0.23147 (3)	0.52325 (3)	0.32511 (4)	0.02585 (15)	
H1	0.246 (4)	0.520 (4)	0.426 (5)	0.039*	
F1A	0.4365 (6)	0.6046 (11)	0.5595 (9)	0.109 (4)	0.464 (8)
F1	0.4744 (9)	0.5657 (7)	0.5371 (13)	0.110 (7)	0.536 (8)
F2A	0.5027 (9)	0.5735 (9)	0.5113 (16)	0.110 (7)	0.464 (8)
F2	0.4384 (7)	0.6600 (7)	0.5192 (10)	0.109 (5)	0.536 (8)
F3A	0.4751 (10)	0.6698 (7)	0.5007 (12)	0.109 (5)	0.464 (8)
F3	0.5061 (5)	0.6352 (9)	0.4725 (8)	0.109 (4)	0.536 (8)
F4	0.4282 (4)	0.6384 (4)	0.1750 (7)	0.129 (4)	
F5	0.3490 (4)	0.5935 (8)	0.1099 (6)	0.190 (7)	
F6	0.4288 (5)	0.5425 (4)	0.1679 (7)	0.137 (4)	
F7	0.1194 (3)	0.7214 (5)	0.4377 (4)	0.117 (3)	
F8	0.0405 (3)	0.6758 (3)	0.3649 (5)	0.087 (2)	
F9	0.0577 (3)	0.7667 (3)	0.3300 (4)	0.0722 (17)	
F10	0.1741 (2)	0.6968 (3)	0.0655 (3)	0.0534 (12)	
F11	0.1048 (3)	0.7579 (3)	0.0734 (3)	0.0630 (14)	
F12	0.0850 (3)	0.6622 (3)	0.0332 (3)	0.0694 (17)	
C1	0.0722 (3)	0.3493 (3)	0.1721 (4)	0.0279 (13)	
C2	0.0639 (3)	0.4093 (3)	0.1313 (4)	0.0285 (13)	
C3	0.0723 (2)	0.4567 (3)	0.1946 (4)	0.0252 (13)	
C4	0.0858 (3)	0.4259 (3)	0.2733 (4)	0.0276 (13)	
C5	0.0854 (3)	0.3593 (3)	0.2592 (4)	0.0284 (13)	
C6	0.0613 (3)	0.2873 (3)	0.1245 (5)	0.0399 (17)	
H6A	0.063316	0.252425	0.163798	0.060*	
H6B	0.091982	0.281264	0.097991	0.060*	
H6C	0.021438	0.288026	0.080787	0.060*	
C7	0.0410 (3)	0.4208 (4)	0.0382 (5)	0.0377 (16)	
H7A	−0.003004	0.419826	0.017583	0.056*	
H7B	0.056238	0.387892	0.009666	0.056*	
H7C	0.054762	0.462383	0.026230	0.056*	
C8	0.0588 (3)	0.5254 (3)	0.1753 (5)	0.0340 (15)	
H8A	0.015918	0.530556	0.143154	0.051*	
H8B	0.082587	0.541203	0.141780	0.051*	
H8C	0.068911	0.549283	0.228180	0.051*	
C9	0.0943 (3)	0.4553 (4)	0.3573 (5)	0.0394 (17)	
H9A	0.097478	0.501394	0.353127	0.059*	
H9B	0.131085	0.438836	0.399139	0.059*	
H9C	0.059806	0.445118	0.374915	0.059*	
C10	0.0858 (3)	0.3111 (4)	0.3250 (5)	0.0396 (17)	
H10A	0.048729	0.314808	0.338548	0.059*	
H10B	0.120489	0.318517	0.376143	0.059*	
H10C	0.088414	0.268562	0.303315	0.059*	
C11	0.2315 (3)	0.2881 (3)	0.2742 (5)	0.0392 (17)	
C12	0.2580 (3)	0.3278 (3)	0.3464 (5)	0.0351 (15)	
C13	0.2977 (3)	0.3702 (3)	0.3269 (5)	0.0320 (15)	
C14	0.2950 (3)	0.3581 (4)	0.2433 (5)	0.0387 (17)	
C15	0.2536 (3)	0.3084 (4)	0.2096 (5)	0.0398 (17)	
C16	0.1953 (3)	0.2293 (4)	0.2722 (7)	0.053 (2)	
H16A	0.163938	0.225434	0.216741	0.079*	
H16B	0.176690	0.231989	0.316107	0.079*	
H16C	0.221698	0.192246	0.282553	0.079*	
C17	0.2496 (4)	0.3238 (4)	0.4304 (5)	0.050 (2)	
H17A	0.288641	0.317533	0.474717	0.075*	
H17B	0.223126	0.288127	0.430502	0.075*	
H17C	0.231464	0.363186	0.441232	0.075*	
C18	0.3429 (3)	0.4092 (4)	0.3919 (6)	0.049 (2)	
H18A	0.322576	0.434577	0.423024	0.073*	
H18B	0.363414	0.437359	0.364152	0.073*	
H18C	0.372367	0.381303	0.431330	0.073*	
C19	0.3359 (4)	0.3871 (5)	0.2017 (7)	0.062 (3)	
H19A	0.338664	0.432828	0.212015	0.093*	
H19B	0.319838	0.379129	0.140729	0.093*	
H19C	0.376000	0.368182	0.225204	0.093*	
C20	0.2392 (4)	0.2778 (5)	0.1243 (6)	0.060 (3)	
H20A	0.258735	0.301474	0.090570	0.089*	
H20B	0.195631	0.277903	0.095305	0.089*	
H20C	0.253939	0.234074	0.131298	0.089*	
C21	0.3109 (3)	0.5558 (3)	0.3272 (5)	0.0298 (14)	
C22	0.3540 (3)	0.5740 (4)	0.4035 (5)	0.0409 (17)	
H22	0.344913	0.571929	0.454317	0.049*	
C23	0.4105 (3)	0.5953 (4)	0.4056 (7)	0.049 (2)	
C24	0.4240 (3)	0.6000 (4)	0.3321 (7)	0.056 (3)	
H24	0.462479	0.614503	0.333896	0.067*	
C25	0.3812 (3)	0.5835 (4)	0.2557 (6)	0.049 (2)	
C26	0.3249 (3)	0.5609 (3)	0.2540 (5)	0.0378 (16)	
H26	0.295972	0.548802	0.201613	0.045*	
C27	0.4564 (4)	0.6116 (4)	0.4879 (7)	0.075 (4)	
C28	0.3955 (4)	0.5882 (5)	0.1769 (8)	0.067 (3)	
C29	0.1829 (3)	0.6014 (3)	0.2877 (4)	0.0241 (12)	
C30	0.1740 (3)	0.6265 (3)	0.2080 (4)	0.0271 (13)	
H30	0.193921	0.607524	0.173506	0.033*	
C31	0.1371 (3)	0.6781 (3)	0.1772 (4)	0.0273 (13)	
C32	0.1080 (3)	0.7072 (3)	0.2261 (4)	0.0314 (14)	
H32	0.082281	0.742376	0.205070	0.038*	
C33	0.1171 (3)	0.6837 (3)	0.3066 (5)	0.0318 (14)	
C34	0.1543 (3)	0.6318 (3)	0.3374 (4)	0.0291 (13)	
H34	0.160344	0.616920	0.393001	0.035*	
C35	0.1250 (3)	0.6995 (3)	0.0884 (5)	0.0351 (15)	
C36	0.0855 (4)	0.7132 (4)	0.3606 (5)	0.048 (2)	

### Bis[3,5-bis(trifluoromethyl)phenyl-2κC1](hydrido-2κH)(iodido-1κI)bis[1,1(η5)-pentamethylcyclopentadienyl]germaniumuranium(Ge—U) (1) Atomic displacement parameters (Å^2^)

**Table:**

	*U*^11^	*U*^22^	*U*^33^	*U*^12^	*U*^13^	*U*^23^
U1	0.01205 (11)	0.02461 (12)	0.02617 (13)	0.00298 (8)	0.00192 (8)	0.00320 (9)
I1	0.0379 (3)	0.0551 (3)	0.0289 (2)	0.0017 (2)	0.00962 (19)	0.0068 (2)
Ge1	0.0192 (3)	0.0290 (3)	0.0281 (3)	−0.0005 (2)	0.0064 (3)	0.0019 (3)
F1A	0.052 (5)	0.174 (14)	0.077 (7)	−0.054 (7)	−0.008 (5)	−0.034 (8)
F1	0.105 (16)	0.094 (8)	0.081 (13)	0.008 (10)	−0.032 (10)	−0.004 (7)
F2A	0.105 (16)	0.094 (8)	0.081 (13)	0.008 (10)	−0.032 (10)	−0.004 (7)
F2	0.126 (15)	0.090 (8)	0.080 (9)	−0.032 (9)	−0.007 (10)	−0.034 (7)
F3A	0.126 (15)	0.090 (8)	0.080 (9)	−0.032 (9)	−0.007 (10)	−0.034 (7)
F3	0.052 (5)	0.174 (14)	0.077 (7)	−0.054 (7)	−0.008 (5)	−0.034 (8)
F4	0.155 (8)	0.091 (5)	0.198 (10)	−0.015 (5)	0.134 (8)	0.022 (6)
F5	0.074 (6)	0.43 (2)	0.077 (6)	−0.021 (8)	0.043 (5)	0.056 (9)
F6	0.210 (11)	0.092 (6)	0.172 (9)	0.036 (6)	0.149 (9)	0.006 (6)
F7	0.087 (5)	0.207 (9)	0.042 (3)	0.078 (5)	0.002 (3)	−0.042 (4)
F8	0.079 (4)	0.100 (5)	0.114 (5)	0.016 (4)	0.073 (4)	0.013 (4)
F9	0.091 (4)	0.065 (4)	0.074 (4)	0.043 (3)	0.045 (3)	0.008 (3)
F10	0.045 (3)	0.079 (4)	0.039 (3)	−0.005 (2)	0.018 (2)	0.013 (2)
F11	0.078 (4)	0.053 (3)	0.055 (3)	0.016 (3)	0.019 (3)	0.024 (2)
F12	0.069 (4)	0.084 (4)	0.034 (3)	−0.047 (3)	−0.010 (2)	0.002 (2)
C1	0.015 (3)	0.024 (3)	0.041 (4)	−0.002 (2)	0.004 (3)	0.000 (3)
C2	0.014 (3)	0.034 (3)	0.032 (3)	0.001 (2)	0.002 (2)	0.001 (3)
C3	0.006 (2)	0.028 (3)	0.035 (3)	0.001 (2)	−0.003 (2)	0.001 (3)
C4	0.010 (3)	0.037 (3)	0.035 (3)	0.007 (2)	0.006 (2)	0.000 (3)
C5	0.012 (3)	0.036 (3)	0.035 (4)	−0.004 (2)	0.005 (2)	0.006 (3)
C6	0.026 (3)	0.033 (4)	0.053 (5)	0.002 (3)	0.003 (3)	−0.005 (3)
C7	0.025 (3)	0.044 (4)	0.032 (4)	0.002 (3)	−0.006 (3)	0.002 (3)
C8	0.018 (3)	0.026 (3)	0.048 (4)	−0.001 (2)	−0.002 (3)	0.000 (3)
C9	0.027 (3)	0.055 (5)	0.037 (4)	0.000 (3)	0.012 (3)	−0.011 (3)
C10	0.029 (4)	0.045 (4)	0.046 (4)	0.000 (3)	0.014 (3)	0.012 (3)
C11	0.023 (3)	0.027 (3)	0.059 (5)	0.002 (3)	0.002 (3)	−0.002 (3)
C12	0.029 (3)	0.025 (3)	0.045 (4)	0.006 (3)	0.005 (3)	0.009 (3)
C13	0.014 (3)	0.027 (3)	0.047 (4)	0.009 (2)	−0.001 (3)	0.008 (3)
C14	0.018 (3)	0.042 (4)	0.057 (5)	0.011 (3)	0.013 (3)	0.009 (3)
C15	0.022 (3)	0.042 (4)	0.051 (4)	0.016 (3)	0.006 (3)	−0.005 (3)
C16	0.029 (4)	0.028 (4)	0.092 (7)	0.008 (3)	0.007 (4)	0.008 (4)
C17	0.045 (5)	0.056 (5)	0.041 (4)	0.013 (4)	0.005 (4)	0.016 (4)
C18	0.022 (3)	0.037 (4)	0.065 (6)	0.005 (3)	−0.015 (3)	0.006 (4)
C19	0.035 (4)	0.072 (6)	0.087 (7)	0.018 (4)	0.032 (5)	0.023 (5)
C20	0.051 (5)	0.063 (6)	0.059 (6)	0.022 (4)	0.010 (4)	−0.018 (5)
C21	0.016 (3)	0.025 (3)	0.042 (4)	0.002 (2)	0.001 (3)	0.000 (3)
C22	0.028 (4)	0.034 (4)	0.050 (5)	0.003 (3)	−0.001 (3)	−0.003 (3)
C23	0.018 (3)	0.035 (4)	0.081 (7)	0.000 (3)	−0.002 (4)	−0.007 (4)
C24	0.017 (3)	0.044 (5)	0.102 (8)	−0.001 (3)	0.013 (4)	0.001 (5)
C25	0.024 (4)	0.046 (5)	0.080 (6)	0.007 (3)	0.022 (4)	0.011 (4)
C26	0.020 (3)	0.032 (4)	0.061 (5)	0.004 (3)	0.013 (3)	0.002 (3)
C27	0.034 (5)	0.054 (6)	0.113 (10)	−0.010 (4)	−0.006 (5)	−0.024 (6)
C28	0.043 (5)	0.071 (7)	0.099 (9)	−0.001 (5)	0.044 (6)	0.009 (6)
C29	0.015 (3)	0.025 (3)	0.033 (3)	−0.002 (2)	0.008 (2)	−0.003 (2)
C30	0.020 (3)	0.030 (3)	0.033 (3)	−0.008 (2)	0.010 (3)	−0.003 (3)
C31	0.020 (3)	0.031 (3)	0.027 (3)	−0.010 (2)	0.003 (2)	−0.001 (2)
C32	0.019 (3)	0.033 (3)	0.037 (4)	−0.002 (3)	0.002 (3)	−0.001 (3)
C33	0.024 (3)	0.031 (3)	0.038 (4)	−0.002 (3)	0.008 (3)	−0.003 (3)
C34	0.023 (3)	0.034 (3)	0.029 (3)	−0.003 (3)	0.007 (3)	0.002 (3)
C35	0.031 (4)	0.035 (4)	0.036 (4)	−0.008 (3)	0.006 (3)	0.009 (3)
C36	0.043 (4)	0.064 (5)	0.045 (5)	0.012 (4)	0.024 (4)	0.003 (4)

### Bis[3,5-bis(trifluoromethyl)phenyl-2κC1](hydrido-2κH)(iodido-1κI)bis[1,1(η5)-pentamethylcyclopentadienyl]germaniumuranium(Ge—U) (1) Geometric parameters (Å, º)

**Table:**

U1—I1	2.9511 (6)	C9—H9C	0.9800
U1—Ge1	3.0428 (7)	C10—H10A	0.9800
U1—C1	2.714 (6)	C10—H10B	0.9800
U1—C2	2.731 (6)	C10—H10C	0.9800
U1—C3	2.738 (6)	C11—C12	1.434 (11)
U1—C4	2.718 (6)	C11—C15	1.420 (12)
U1—C5	2.712 (6)	C11—C16	1.502 (10)
U1—C11	2.686 (7)	C12—C13	1.413 (10)
U1—C12	2.707 (7)	C12—C17	1.494 (11)
U1—C13	2.756 (6)	C13—C14	1.411 (11)
U1—C14	2.735 (6)	C13—C18	1.492 (10)
U1—C15	2.674 (7)	C14—C15	1.418 (11)
Ge1—H1	1.61 (8)	C14—C19	1.501 (11)
Ge1—C21	1.990 (6)	C15—C20	1.505 (12)
Ge1—C29	1.988 (6)	C16—H16A	0.9800
F1A—C27	1.440 (14)	C16—H16B	0.9800
F1—C27	1.252 (14)	C16—H16C	0.9800
F2A—C27	1.307 (15)	C17—H17A	0.9800
F2—C27	1.285 (13)	C17—H17B	0.9800
F3A—C27	1.298 (13)	C17—H17C	0.9800
F3—C27	1.379 (12)	C18—H18A	0.9800
F4—C28	1.318 (10)	C18—H18B	0.9800
F5—C28	1.287 (12)	C18—H18C	0.9800
F6—C28	1.287 (10)	C19—H19A	0.9800
F7—C36	1.285 (10)	C19—H19B	0.9800
F8—C36	1.348 (11)	C19—H19C	0.9800
F9—C36	1.321 (10)	C20—H20A	0.9800
F10—C35	1.341 (9)	C20—H20B	0.9800
F11—C35	1.315 (9)	C20—H20C	0.9800
F12—C35	1.336 (8)	C21—C22	1.396 (10)
C1—C2	1.422 (9)	C21—C26	1.382 (11)
C1—C5	1.406 (10)	C22—H22	0.9500
C1—C6	1.511 (9)	C22—C23	1.397 (11)
C2—C3	1.426 (9)	C23—C24	1.382 (15)
C2—C7	1.494 (10)	C23—C27	1.484 (13)
C3—C4	1.411 (9)	C24—H24	0.9500
C3—C8	1.497 (9)	C24—C25	1.386 (14)
C4—C5	1.425 (10)	C25—C26	1.404 (10)
C4—C9	1.494 (10)	C25—C28	1.477 (14)
C5—C10	1.502 (9)	C26—H26	0.9500
C6—H6A	0.9800	C29—C30	1.389 (9)
C6—H6B	0.9800	C29—C34	1.397 (9)
C6—H6C	0.9800	C30—H30	0.9500
C7—H7A	0.9800	C30—C31	1.382 (9)
C7—H7B	0.9800	C31—C32	1.383 (9)
C7—H7C	0.9800	C31—C35	1.493 (9)
C8—H8A	0.9800	C32—H32	0.9500
C8—H8B	0.9800	C32—C33	1.388 (10)
C8—H8C	0.9800	C33—C34	1.391 (9)
C9—H9A	0.9800	C33—C36	1.492 (10)
C9—H9B	0.9800	C34—H34	0.9500
			
I1—U1—Ge1	88.063 (19)	H10A—C10—H10B	109.5
C1—U1—I1	104.96 (15)	H10A—C10—H10C	109.5
C1—U1—Ge1	132.39 (14)	H10B—C10—H10C	109.5
C1—U1—C2	30.28 (19)	C12—C11—U1	75.4 (4)
C1—U1—C3	49.91 (18)	C12—C11—C16	125.8 (8)
C1—U1—C4	49.9 (2)	C15—C11—U1	74.2 (4)
C1—U1—C13	137.69 (19)	C15—C11—C12	107.6 (6)
C1—U1—C14	133.0 (2)	C15—C11—C16	125.9 (8)
C2—U1—I1	81.46 (14)	C16—C11—U1	123.9 (5)
C2—U1—Ge1	113.68 (14)	C11—C12—U1	73.8 (4)
C2—U1—C3	30.23 (19)	C11—C12—C17	127.4 (7)
C2—U1—C13	167.88 (19)	C13—C12—U1	76.9 (4)
C2—U1—C14	148.2 (2)	C13—C12—C11	108.0 (7)
C3—U1—I1	90.27 (14)	C13—C12—C17	124.5 (7)
C3—U1—Ge1	85.29 (13)	C17—C12—U1	118.5 (5)
C3—U1—C13	154.3 (2)	C12—C13—U1	73.1 (4)
C4—U1—I1	120.04 (14)	C12—C13—C18	123.2 (7)
C4—U1—Ge1	83.71 (14)	C14—C13—U1	74.3 (4)
C4—U1—C2	49.8 (2)	C14—C13—C12	107.9 (6)
C4—U1—C3	29.97 (19)	C14—C13—C18	127.6 (7)
C4—U1—C13	128.2 (2)	C18—C13—U1	128.7 (4)
C4—U1—C14	156.4 (2)	C13—C14—U1	76.0 (4)
C5—U1—I1	131.23 (14)	C13—C14—C15	108.9 (7)
C5—U1—Ge1	111.14 (15)	C13—C14—C19	124.9 (8)
C5—U1—C1	30.0 (2)	C15—C14—U1	72.5 (4)
C5—U1—C2	49.8 (2)	C15—C14—C19	125.7 (8)
C5—U1—C3	49.87 (19)	C19—C14—U1	124.3 (5)
C5—U1—C4	30.4 (2)	C11—C15—U1	75.1 (4)
C5—U1—C13	121.2 (2)	C11—C15—C20	125.2 (8)
C5—U1—C14	136.3 (2)	C14—C15—U1	77.2 (4)
C11—U1—I1	120.78 (18)	C14—C15—C11	107.6 (7)
C11—U1—Ge1	123.07 (16)	C14—C15—C20	127.0 (8)
C11—U1—C1	89.4 (2)	C20—C15—U1	118.2 (5)
C11—U1—C2	118.1 (2)	C11—C16—H16A	109.5
C11—U1—C3	135.7 (2)	C11—C16—H16B	109.5
C11—U1—C4	113.0 (2)	C11—C16—H16C	109.5
C11—U1—C5	86.5 (2)	H16A—C16—H16B	109.5
C11—U1—C12	30.8 (2)	H16A—C16—H16C	109.5
C11—U1—C13	50.1 (2)	H16B—C16—H16C	109.5
C11—U1—C14	50.0 (2)	C12—C17—H17A	109.5
C12—U1—I1	132.74 (16)	C12—C17—H17B	109.5
C12—U1—Ge1	92.87 (16)	C12—C17—H17C	109.5
C12—U1—C1	108.7 (2)	H17A—C17—H17B	109.5
C12—U1—C2	138.7 (2)	H17A—C17—H17C	109.5
C12—U1—C3	136.9 (2)	H17B—C17—H17C	109.5
C12—U1—C4	107.0 (2)	C13—C18—H18A	109.5
C12—U1—C5	92.1 (2)	C13—C18—H18B	109.5
C12—U1—C13	30.0 (2)	C13—C18—H18C	109.5
C12—U1—C14	49.6 (2)	H18A—C18—H18B	109.5
C13—U1—I1	106.56 (15)	H18A—C18—H18C	109.5
C13—U1—Ge1	76.24 (14)	H18B—C18—H18C	109.5
C14—U1—I1	83.15 (17)	C14—C19—H19A	109.5
C14—U1—Ge1	93.36 (17)	C14—C19—H19B	109.5
C14—U1—C3	173.3 (2)	C14—C19—H19C	109.5
C14—U1—C13	29.8 (2)	H19A—C19—H19B	109.5
C15—U1—I1	90.39 (18)	H19A—C19—H19C	109.5
C15—U1—Ge1	123.18 (17)	H19B—C19—H19C	109.5
C15—U1—C1	102.7 (2)	C15—C20—H20A	109.5
C15—U1—C2	122.2 (2)	C15—C20—H20B	109.5
C15—U1—C3	151.5 (2)	C15—C20—H20C	109.5
C15—U1—C4	141.5 (2)	H20A—C20—H20B	109.5
C15—U1—C5	111.9 (2)	H20A—C20—H20C	109.5
C15—U1—C11	30.7 (3)	H20B—C20—H20C	109.5
C15—U1—C12	50.7 (2)	C22—C21—Ge1	120.2 (6)
C15—U1—C13	50.1 (2)	C26—C21—Ge1	121.3 (5)
C15—U1—C14	30.4 (2)	C26—C21—C22	118.5 (7)
U1—Ge1—H1	117 (3)	C21—C22—H22	119.8
C21—Ge1—U1	118.5 (2)	C21—C22—C23	120.4 (8)
C21—Ge1—H1	97 (3)	C23—C22—H22	119.8
C29—Ge1—U1	116.73 (18)	C22—C23—C27	119.4 (9)
C29—Ge1—H1	105 (3)	C24—C23—C22	120.6 (8)
C29—Ge1—C21	99.2 (2)	C24—C23—C27	120.0 (8)
C2—C1—U1	75.5 (3)	C23—C24—H24	120.2
C2—C1—C6	123.0 (6)	C23—C24—C25	119.5 (7)
C5—C1—U1	74.9 (3)	C25—C24—H24	120.2
C5—C1—C2	108.3 (6)	C24—C25—C26	119.7 (9)
C5—C1—C6	128.4 (6)	C24—C25—C28	119.7 (8)
C6—C1—U1	120.8 (4)	C26—C25—C28	120.5 (9)
C1—C2—U1	74.2 (3)	C21—C26—C25	121.2 (8)
C1—C2—C3	107.7 (6)	C21—C26—H26	119.4
C1—C2—C7	126.4 (6)	C25—C26—H26	119.4
C3—C2—U1	75.1 (3)	F1A—C27—C23	114.3 (9)
C3—C2—C7	125.2 (6)	F1—C27—F2	115.5 (13)
C7—C2—U1	123.9 (4)	F1—C27—F3	106.4 (11)
C2—C3—U1	74.6 (3)	F1—C27—C23	114.5 (12)
C2—C3—C8	123.6 (6)	F2A—C27—F1A	99.3 (12)
C4—C3—U1	74.2 (3)	F2A—C27—C23	114.3 (14)
C4—C3—C2	107.8 (6)	F2—C27—F3	102.2 (10)
C4—C3—C8	127.9 (6)	F2—C27—C23	109.0 (10)
C8—C3—U1	124.4 (4)	F3A—C27—F1A	98.6 (11)
C3—C4—U1	75.8 (3)	F3A—C27—F2A	109.2 (12)
C3—C4—C5	108.2 (6)	F3A—C27—C23	118.4 (12)
C3—C4—C9	127.7 (6)	F3—C27—C23	108.1 (10)
C5—C4—U1	74.6 (3)	F4—C28—C25	113.0 (10)
C5—C4—C9	124.0 (6)	F5—C28—F4	104.1 (11)
C9—C4—U1	119.6 (4)	F5—C28—C25	113.8 (7)
C1—C5—U1	75.1 (3)	F6—C28—F4	102.3 (8)
C1—C5—C4	108.0 (6)	F6—C28—F5	109.8 (12)
C1—C5—C10	127.5 (6)	F6—C28—C25	112.9 (9)
C4—C5—U1	75.0 (3)	C30—C29—Ge1	120.3 (5)
C4—C5—C10	123.3 (6)	C30—C29—C34	117.1 (6)
C10—C5—U1	126.0 (4)	C34—C29—Ge1	122.5 (5)
C1—C6—H6A	109.5	C29—C30—H30	118.9
C1—C6—H6B	109.5	C31—C30—C29	122.2 (6)
C1—C6—H6C	109.5	C31—C30—H30	118.9
H6A—C6—H6B	109.5	C30—C31—C32	120.4 (6)
H6A—C6—H6C	109.5	C30—C31—C35	119.9 (6)
H6B—C6—H6C	109.5	C32—C31—C35	119.6 (6)
C2—C7—H7A	109.5	C31—C32—H32	120.8
C2—C7—H7B	109.5	C31—C32—C33	118.5 (6)
C2—C7—H7C	109.5	C33—C32—H32	120.8
H7A—C7—H7B	109.5	C32—C33—C34	121.0 (6)
H7A—C7—H7C	109.5	C32—C33—C36	120.0 (7)
H7B—C7—H7C	109.5	C34—C33—C36	119.0 (7)
C3—C8—H8A	109.5	C29—C34—H34	119.6
C3—C8—H8B	109.5	C33—C34—C29	120.8 (6)
C3—C8—H8C	109.5	C33—C34—H34	119.6
H8A—C8—H8B	109.5	F10—C35—C31	112.4 (6)
H8A—C8—H8C	109.5	F11—C35—F10	106.2 (6)
H8B—C8—H8C	109.5	F11—C35—F12	107.1 (6)
C4—C9—H9A	109.5	F11—C35—C31	114.3 (7)
C4—C9—H9B	109.5	F12—C35—F10	104.7 (6)
C4—C9—H9C	109.5	F12—C35—C31	111.5 (5)
H9A—C9—H9B	109.5	F7—C36—F8	105.1 (8)
H9A—C9—H9C	109.5	F7—C36—F9	109.7 (8)
H9B—C9—H9C	109.5	F7—C36—C33	113.6 (7)
C5—C10—H10A	109.5	F8—C36—C33	110.8 (7)
C5—C10—H10B	109.5	F9—C36—F8	102.8 (7)
C5—C10—H10C	109.5	F9—C36—C33	114.0 (7)

### Bis[3,5-bis(trifluoromethyl)phenyl-2κC1](fluorido-1κI)(hydrido-2κH)bis[1,1(η5)-pentamethylcyclopentadienyl]germaniumuranium(Ge—U) (2) Crystal data

**Table:**

[GeU(C_10_H_15_)_2_(C_8_H_3_F_6_)_2_FH]	*F*(000) = 4016
*M**_r_* = 1041.30	*D*_x_ = 1.844 Mg m^−^^3^
Monoclinic, *C*2/*c*	Mo *K*α radiation, λ = 0.71073 Å
*a* = 34.160 (3) Å	Cell parameters from 9918 reflections
*b* = 13.5237 (11) Å	θ = 2.3–23.3°
*c* = 16.2986 (13) Å	µ = 5.21 mm^−^^1^
β = 95.028 (2)°	*T* = 100 K
*V* = 7500.4 (10) Å^3^	Prism, clear dark red
*Z* = 8	0.10 × 0.10 × 0.08 mm

### Bis[3,5-bis(trifluoromethyl)phenyl-2κC1](fluorido-1κI)(hydrido-2κH)bis[1,1(η5)-pentamethylcyclopentadienyl]germaniumuranium(Ge—U) (2) Data collection

**Table:**

Bruker APEXII CCD diffractometer	5563 reflections with *I* > 2σ(*I*)
φ and ω scans	*R*_int_ = 0.081
Absorption correction: multi-scan (SADABS; Bruker, 2015)	θ_max_ = 26.5°, θ_min_ = 1.6°
*T*_min_ = 0.501, *T*_max_ = 0.562	*h* = −42→42
65643 measured reflections	*k* = −16→16
7720 independent reflections	*l* = −20→20

### Bis[3,5-bis(trifluoromethyl)phenyl-2κC1](fluorido-1κI)(hydrido-2κH)bis[1,1(η5)-pentamethylcyclopentadienyl]germaniumuranium(Ge—U) (2) Refinement

**Table:**

Refinement on *F*^2^	Primary atom site location: dual
Least-squares matrix: full	Secondary atom site location: difference Fourier map
*R*[*F*^2^ > 2σ(*F*^2^)] = 0.041	Hydrogen site location: inferred from neighbouring sites
*wR*(*F*^2^) = 0.094	H-atom parameters constrained
*S* = 1.05	*w* = 1/[σ^2^(*F*_o_^2^) + (0.0441*P*)^2^ + 21.6495*P*] where *P* = (*F*_o_^2^ + 2*F*_c_^2^)/3
7720 reflections	(Δ/σ)_max_ = 0.001
492 parameters	Δρ_max_ = 1.68 e Å^−^^3^
0 restraints	Δρ_min_ = −0.78 e Å^−^^3^

### Bis[3,5-bis(trifluoromethyl)phenyl-2κC1](fluorido-1κI)(hydrido-2κH)bis[1,1(η5)-pentamethylcyclopentadienyl]germaniumuranium(Ge—U) (2) Special details

**Table:**

Geometry. All esds (except the esd in the dihedral angle between two l.s. planes) are estimated using the full covariance matrix. The cell esds are taken into account individually in the estimation of esds in distances, angles and torsion angles; correlations between esds in cell parameters are only used when they are defined by crystal symmetry. An approximate (isotropic) treatment of cell esds is used for estimating esds involving l.s. planes.

### Bis[3,5-bis(trifluoromethyl)phenyl-2κC1](fluorido-1κI)(hydrido-2κH)bis[1,1(η5)-pentamethylcyclopentadienyl]germaniumuranium(Ge—U) (2) Fractional atomic coordinates and isotropic or equivalent isotropic displacement parameters (Å^2^)

**Table:**

	*x*	*y*	*z*	*U*_iso_*/*U*_eq_	Occ. (<1)
U1	0.37698 (2)	0.91030 (2)	0.40571 (2)	0.03137 (8)	
Ge1	0.34983 (2)	0.70121 (4)	0.35878 (4)	0.02943 (15)	
F1	0.38083 (14)	0.9430 (4)	0.2718 (3)	0.0798 (14)	
F1A	0.4259 (6)	0.6888 (19)	0.0809 (14)	0.059 (7)*	0.25
F2	0.36774 (11)	0.6810 (3)	0.6963 (2)	0.0528 (10)	
F2A	0.4111 (7)	0.5508 (19)	0.0457 (14)	0.070 (6)*	0.25
F3	0.30596 (11)	0.7033 (3)	0.6925 (2)	0.0527 (10)	
F3A	0.4673 (6)	0.5837 (17)	0.0875 (12)	0.064 (5)*	0.25
F4	0.33071 (13)	0.5681 (3)	0.7388 (2)	0.0590 (11)	
F5	0.2643 (2)	0.3420 (4)	0.5447 (4)	0.125 (3)	
F6	0.30011 (16)	0.3142 (4)	0.4505 (5)	0.128 (3)	
F7	0.24878 (18)	0.3913 (4)	0.4284 (4)	0.106 (2)	
F8	0.42298 (13)	0.3341 (3)	0.3844 (3)	0.0679 (13)	
F9	0.45845 (18)	0.3155 (4)	0.2866 (3)	0.102 (2)	
F10	0.47779 (16)	0.4025 (4)	0.3882 (4)	0.103 (2)	
F11	0.39263 (19)	0.6235 (7)	0.0486 (3)	0.088 (2)	0.75
F12	0.4469 (3)	0.6813 (7)	0.0902 (4)	0.089 (3)	0.75
F13	0.4411 (4)	0.5303 (5)	0.0554 (4)	0.102 (3)	0.75
C1	0.30338 (17)	0.9809 (5)	0.3689 (4)	0.0377 (15)	
C2	0.30009 (17)	0.9202 (5)	0.4381 (4)	0.0359 (14)	
C3	0.32110 (17)	0.9643 (5)	0.5051 (4)	0.0349 (15)	
C4	0.33680 (19)	1.0537 (5)	0.4790 (5)	0.0451 (17)	
C5	0.3263 (2)	1.0616 (5)	0.3930 (5)	0.0456 (18)	
C6	0.28286 (19)	0.9627 (6)	0.2847 (4)	0.057 (2)	
H6A	0.302274	0.944506	0.246500	0.086*	
H6B	0.269101	1.022952	0.265059	0.086*	
H6C	0.263849	0.908854	0.287715	0.086*	
C7	0.27336 (17)	0.8335 (5)	0.4455 (5)	0.0482 (18)	
H7A	0.264189	0.809488	0.390379	0.072*	
H7B	0.250742	0.853633	0.474574	0.072*	
H7C	0.287652	0.780550	0.476327	0.072*	
C8	0.3214 (2)	0.9282 (6)	0.5923 (4)	0.0526 (19)	
H8A	0.330927	0.859888	0.595617	0.079*	
H8B	0.294675	0.930990	0.609746	0.079*	
H8C	0.338746	0.970290	0.628403	0.079*	
C9	0.3541 (2)	1.1334 (6)	0.5340 (6)	0.077 (3)	
H9A	0.369275	1.103688	0.581581	0.115*	
H9B	0.333002	1.174235	0.553025	0.115*	
H9C	0.371460	1.174702	0.503573	0.115*	
C10	0.3358 (3)	1.1463 (6)	0.3375 (6)	0.076 (3)	
H10A	0.356864	1.186643	0.365227	0.114*	
H10B	0.312351	1.187129	0.325362	0.114*	
H10C	0.344460	1.119977	0.286069	0.114*	
C11	0.45358 (18)	0.9564 (6)	0.4048 (5)	0.0511 (19)	
C12	0.45269 (17)	0.8548 (6)	0.4005 (4)	0.0464 (18)	
C13	0.44244 (16)	0.8168 (5)	0.4758 (4)	0.0367 (15)	
C14	0.43624 (16)	0.8966 (5)	0.5274 (4)	0.0374 (15)	
C15	0.44352 (18)	0.9842 (5)	0.4837 (5)	0.0457 (17)	
C16	0.4660 (2)	1.0261 (9)	0.3402 (6)	0.110 (4)	
H16A	0.459523	0.997306	0.285517	0.165*	
H16B	0.494410	1.037220	0.348837	0.165*	
H16C	0.452177	1.089153	0.344149	0.165*	
C17	0.4655 (2)	0.7930 (8)	0.3298 (5)	0.090 (3)	
H17A	0.456631	0.724666	0.335665	0.135*	
H17B	0.494219	0.794334	0.330707	0.135*	
H17C	0.453917	0.820165	0.277461	0.135*	
C18	0.4437 (2)	0.7100 (5)	0.5018 (5)	0.065 (2)	
H18A	0.426927	0.700429	0.546950	0.097*	
H18B	0.470798	0.691613	0.520396	0.097*	
H18C	0.434235	0.668309	0.455030	0.097*	
C19	0.4270 (2)	0.8889 (7)	0.6161 (4)	0.064 (2)	
H19A	0.420021	0.954337	0.636099	0.096*	
H19B	0.450102	0.863895	0.649682	0.096*	
H19C	0.404921	0.843410	0.620074	0.096*	
C20	0.4470 (2)	1.0879 (6)	0.5166 (7)	0.084 (3)	
H20A	0.430763	1.132280	0.480334	0.126*	
H20B	0.474508	1.109160	0.518893	0.126*	
H20C	0.438032	1.089872	0.572071	0.126*	
C21	0.33441 (17)	0.6153 (4)	0.4498 (4)	0.0304 (13)	
C22	0.34129 (16)	0.6465 (4)	0.5310 (4)	0.0298 (13)	
H22	0.355005	0.706794	0.542250	0.036*	
C23	0.32877 (16)	0.5925 (4)	0.5960 (4)	0.0298 (13)	
C24	0.30822 (17)	0.5052 (4)	0.5809 (4)	0.0355 (15)	
H24	0.299070	0.468129	0.624861	0.043*	
C25	0.30132 (17)	0.4732 (4)	0.5005 (4)	0.0331 (14)	
C26	0.31381 (16)	0.5275 (4)	0.4362 (4)	0.0325 (14)	
H26	0.308182	0.504397	0.381438	0.039*	
C27	0.33361 (18)	0.6341 (5)	0.6810 (4)	0.0374 (15)	
C28	0.2795 (2)	0.3801 (5)	0.4824 (5)	0.0458 (17)	
C29	0.38704 (17)	0.6139 (4)	0.3047 (4)	0.0322 (14)	
C30	0.40422 (16)	0.5301 (4)	0.3407 (4)	0.0313 (13)	
H30	0.399218	0.513209	0.395424	0.038*	
C31	0.42879 (17)	0.4699 (4)	0.2982 (4)	0.0328 (14)	
C32	0.43653 (18)	0.4931 (5)	0.2190 (4)	0.0353 (14)	
H32	0.453532	0.452673	0.190272	0.042*	
C33	0.41927 (19)	0.5758 (4)	0.1818 (4)	0.0345 (14)	
C34	0.39482 (17)	0.6349 (5)	0.2230 (4)	0.0338 (14)	
H34	0.382949	0.691027	0.196017	0.041*	
C35	0.4467 (2)	0.3801 (5)	0.3383 (4)	0.0454 (17)	
C36	0.4265 (2)	0.6010 (5)	0.0957 (4)	0.0425 (16)	
C37	0.30396 (19)	0.6882 (5)	0.2750 (4)	0.0440 (16)	
H37A	0.280389	0.714353	0.297535	0.066*	
H37B	0.299901	0.618264	0.260811	0.066*	
H37C	0.309036	0.725430	0.225470	0.066*	

### Bis[3,5-bis(trifluoromethyl)phenyl-2κC1](fluorido-1κI)(hydrido-2κH)bis[1,1(η5)-pentamethylcyclopentadienyl]germaniumuranium(Ge—U) (2) Atomic displacement parameters (Å^2^)

**Table:**

	*U*^11^	*U*^22^	*U*^33^	*U*^12^	*U*^13^	*U*^23^
U1	0.02538 (11)	0.02903 (13)	0.04074 (15)	0.00002 (10)	0.00874 (9)	−0.00207 (11)
Ge1	0.0341 (3)	0.0281 (3)	0.0267 (3)	0.0022 (3)	0.0062 (3)	−0.0023 (3)
F1	0.068 (3)	0.100 (4)	0.073 (3)	0.009 (3)	0.013 (3)	0.026 (3)
F2	0.055 (2)	0.060 (3)	0.041 (2)	−0.015 (2)	−0.0021 (19)	−0.0006 (19)
F3	0.062 (2)	0.059 (3)	0.037 (2)	0.007 (2)	0.0064 (19)	−0.0077 (19)
F4	0.080 (3)	0.059 (3)	0.037 (2)	−0.015 (2)	−0.001 (2)	0.0139 (19)
F5	0.205 (7)	0.088 (4)	0.089 (4)	−0.099 (5)	0.049 (4)	−0.022 (3)
F6	0.082 (4)	0.048 (3)	0.262 (8)	−0.015 (3)	0.061 (5)	−0.070 (4)
F7	0.100 (4)	0.062 (3)	0.147 (5)	−0.034 (3)	−0.036 (4)	−0.004 (3)
F8	0.081 (3)	0.050 (3)	0.077 (3)	0.022 (2)	0.034 (3)	0.026 (2)
F9	0.168 (5)	0.081 (4)	0.064 (3)	0.086 (4)	0.049 (3)	0.023 (3)
F10	0.073 (3)	0.101 (4)	0.127 (5)	0.010 (3)	−0.035 (3)	0.042 (4)
F11	0.061 (4)	0.172 (8)	0.032 (3)	0.016 (5)	0.009 (3)	0.036 (4)
F12	0.114 (7)	0.113 (7)	0.041 (4)	−0.080 (6)	0.015 (4)	0.012 (4)
F13	0.217 (10)	0.063 (5)	0.037 (4)	0.080 (6)	0.062 (5)	0.019 (3)
C1	0.030 (3)	0.048 (4)	0.037 (4)	0.014 (3)	0.013 (3)	0.014 (3)
C2	0.030 (3)	0.039 (4)	0.041 (4)	0.004 (3)	0.014 (3)	0.002 (3)
C3	0.038 (3)	0.038 (4)	0.031 (4)	0.013 (3)	0.015 (3)	0.002 (3)
C4	0.037 (3)	0.030 (3)	0.070 (5)	0.005 (3)	0.013 (3)	−0.008 (3)
C5	0.045 (4)	0.039 (4)	0.056 (5)	0.010 (3)	0.021 (3)	0.017 (3)
C6	0.038 (4)	0.097 (6)	0.038 (4)	0.022 (4)	0.009 (3)	0.017 (4)
C7	0.028 (3)	0.051 (4)	0.069 (5)	0.000 (3)	0.021 (3)	0.004 (4)
C8	0.057 (4)	0.068 (5)	0.035 (4)	0.022 (4)	0.019 (3)	0.003 (3)
C9	0.064 (5)	0.059 (5)	0.108 (8)	0.007 (4)	0.012 (5)	−0.043 (5)
C10	0.093 (6)	0.039 (5)	0.102 (7)	0.019 (4)	0.048 (6)	0.036 (5)
C11	0.026 (3)	0.081 (6)	0.046 (5)	−0.016 (4)	0.002 (3)	0.021 (4)
C12	0.023 (3)	0.073 (6)	0.044 (4)	−0.001 (3)	0.007 (3)	−0.011 (4)
C13	0.021 (3)	0.038 (4)	0.051 (4)	0.005 (3)	0.002 (3)	−0.002 (3)
C14	0.025 (3)	0.053 (4)	0.034 (4)	0.003 (3)	0.001 (3)	−0.006 (3)
C15	0.032 (3)	0.034 (4)	0.069 (5)	−0.009 (3)	−0.006 (3)	−0.002 (4)
C16	0.059 (5)	0.167 (11)	0.102 (8)	−0.059 (6)	−0.010 (5)	0.080 (8)
C17	0.037 (4)	0.169 (10)	0.067 (6)	0.005 (5)	0.018 (4)	−0.049 (6)
C18	0.045 (4)	0.041 (4)	0.103 (7)	0.010 (3)	−0.021 (4)	0.003 (4)
C19	0.042 (4)	0.112 (7)	0.036 (4)	0.004 (4)	−0.003 (3)	−0.001 (4)
C20	0.047 (4)	0.047 (5)	0.151 (9)	−0.004 (4)	−0.033 (5)	−0.021 (5)
C21	0.031 (3)	0.029 (3)	0.032 (3)	0.002 (2)	0.006 (3)	0.000 (3)
C22	0.032 (3)	0.026 (3)	0.032 (3)	0.000 (2)	0.005 (3)	−0.002 (3)
C23	0.029 (3)	0.028 (3)	0.033 (3)	0.000 (3)	0.004 (2)	0.002 (3)
C24	0.032 (3)	0.033 (3)	0.043 (4)	0.001 (3)	0.011 (3)	0.006 (3)
C25	0.035 (3)	0.023 (3)	0.042 (4)	0.002 (3)	0.011 (3)	−0.005 (3)
C26	0.037 (3)	0.029 (3)	0.032 (3)	0.003 (3)	0.008 (3)	−0.006 (3)
C27	0.039 (3)	0.035 (4)	0.037 (4)	−0.004 (3)	−0.003 (3)	0.007 (3)
C28	0.053 (4)	0.026 (3)	0.059 (5)	−0.003 (3)	0.012 (4)	−0.004 (3)
C29	0.039 (3)	0.025 (3)	0.034 (4)	−0.004 (3)	0.010 (3)	−0.011 (3)
C30	0.038 (3)	0.034 (3)	0.023 (3)	−0.001 (3)	0.009 (3)	−0.001 (3)
C31	0.036 (3)	0.033 (3)	0.030 (3)	0.000 (3)	0.008 (3)	0.000 (3)
C32	0.040 (3)	0.035 (4)	0.033 (4)	0.004 (3)	0.012 (3)	−0.006 (3)
C33	0.047 (4)	0.030 (3)	0.028 (3)	−0.003 (3)	0.009 (3)	−0.001 (3)
C34	0.041 (3)	0.030 (3)	0.031 (4)	0.004 (3)	0.009 (3)	−0.002 (3)
C35	0.050 (4)	0.053 (4)	0.035 (4)	0.014 (3)	0.011 (3)	0.006 (3)
C36	0.064 (5)	0.031 (4)	0.034 (4)	0.000 (4)	0.018 (3)	−0.004 (3)
C37	0.049 (4)	0.046 (4)	0.037 (4)	0.010 (3)	0.002 (3)	−0.004 (3)

### Bis[3,5-bis(trifluoromethyl)phenyl-2κC1](fluorido-1κI)(hydrido-2κH)bis[1,1(η5)-pentamethylcyclopentadienyl]germaniumuranium(Ge—U) (2) Geometric parameters (Å, º)

**Table:**

U1—F1	2.243 (5)	C10—H10B	0.9800
U1—C5	2.676 (6)	C10—H10C	0.9800
U1—C11	2.691 (6)	C11—C12	1.375 (11)
U1—C15	2.697 (6)	C11—C15	1.411 (10)
U1—C12	2.701 (6)	C11—C16	1.501 (10)
U1—C1	2.707 (6)	C12—C13	1.403 (9)
U1—C3	2.710 (5)	C12—C17	1.519 (10)
U1—C4	2.712 (6)	C13—C14	1.396 (9)
U1—C14	2.713 (6)	C13—C18	1.505 (9)
U1—C2	2.727 (6)	C14—C15	1.415 (9)
U1—C13	2.731 (6)	C14—C19	1.510 (9)
U1—Ge1	3.0524 (7)	C15—C20	1.502 (10)
Ge1—C21	1.991 (6)	C16—H16A	0.9800
Ge1—C37	1.994 (6)	C16—H16B	0.9800
Ge1—C29	1.996 (6)	C16—H16C	0.9800
F1A—C36	1.21 (2)	C17—H17A	0.9800
F2—C27	1.331 (7)	C17—H17B	0.9800
F2A—C36	1.15 (2)	C17—H17C	0.9800
F3—C27	1.354 (7)	C18—H18A	0.9800
F3A—C36	1.43 (2)	C18—H18B	0.9800
F4—C27	1.308 (7)	C18—H18C	0.9800
F5—C28	1.288 (8)	C19—H19A	0.9800
F6—C28	1.274 (8)	C19—H19B	0.9800
F7—C28	1.319 (9)	C19—H19C	0.9800
F8—C35	1.309 (8)	C20—H20A	0.9800
F9—C35	1.302 (8)	C20—H20B	0.9800
F10—C35	1.315 (8)	C20—H20C	0.9800
F11—C36	1.367 (9)	C21—C26	1.388 (8)
F12—C36	1.296 (9)	C21—C22	1.389 (8)
F13—C36	1.284 (8)	C22—C23	1.386 (8)
C1—C5	1.381 (9)	C22—H22	0.9500
C1—C2	1.407 (8)	C23—C24	1.384 (8)
C1—C6	1.506 (9)	C23—C27	1.491 (9)
C2—C3	1.388 (9)	C24—C25	1.380 (8)
C2—C7	1.498 (9)	C24—H24	0.9500
C3—C4	1.403 (9)	C25—C26	1.377 (8)
C3—C8	1.502 (9)	C25—C28	1.479 (9)
C4—C5	1.419 (10)	C26—H26	0.9500
C4—C9	1.491 (10)	C29—C30	1.383 (8)
C5—C10	1.512 (9)	C29—C34	1.410 (8)
C6—H6A	0.9800	C30—C31	1.397 (8)
C6—H6B	0.9800	C30—H30	0.9500
C6—H6C	0.9800	C31—C32	1.377 (8)
C7—H7A	0.9800	C31—C35	1.485 (9)
C7—H7B	0.9800	C32—C33	1.379 (8)
C7—H7C	0.9800	C32—H32	0.9500
C8—H8A	0.9800	C33—C34	1.374 (8)
C8—H8B	0.9800	C33—C36	1.486 (9)
C8—H8C	0.9800	C34—H34	0.9500
C9—H9A	0.9800	C37—H37A	0.9800
C9—H9B	0.9800	C37—H37B	0.9800
C9—H9C	0.9800	C37—H37C	0.9800
C10—H10A	0.9800		
			
F1—U1—C5	82.3 (2)	C12—C11—C15	107.8 (6)
F1—U1—C11	78.98 (19)	C12—C11—C16	126.7 (9)
C5—U1—C11	116.4 (2)	C15—C11—C16	125.3 (9)
F1—U1—C15	105.5 (2)	C12—C11—U1	75.6 (4)
C5—U1—C15	105.4 (2)	C15—C11—U1	75.0 (3)
C11—U1—C15	30.4 (2)	C16—C11—U1	118.9 (5)
F1—U1—C12	83.48 (19)	C11—C12—C13	109.2 (6)
C5—U1—C12	145.5 (2)	C11—C12—C17	125.5 (8)
C11—U1—C12	29.5 (2)	C13—C12—C17	124.9 (8)
C15—U1—C12	49.3 (2)	C11—C12—U1	74.8 (4)
F1—U1—C1	81.23 (17)	C13—C12—U1	76.2 (3)
C5—U1—C1	29.7 (2)	C17—C12—U1	121.1 (4)
C11—U1—C1	143.3 (2)	C14—C13—C12	107.8 (6)
C15—U1—C1	134.6 (2)	C14—C13—C18	125.1 (7)
C12—U1—C1	164.6 (2)	C12—C13—C18	126.5 (7)
F1—U1—C3	129.29 (18)	C14—C13—U1	74.5 (3)
C5—U1—C3	49.87 (19)	C12—C13—U1	73.9 (4)
C11—U1—C3	132.6 (2)	C18—C13—U1	124.4 (4)
C15—U1—C3	103.1 (2)	C13—C14—C15	107.5 (6)
C12—U1—C3	145.1 (2)	C13—C14—C19	125.4 (7)
C1—U1—C3	49.31 (18)	C15—C14—C19	126.9 (7)
F1—U1—C4	111.3 (2)	C13—C14—U1	75.8 (3)
C5—U1—C4	30.5 (2)	C15—C14—U1	74.2 (4)
C11—U1—C4	111.5 (2)	C19—C14—U1	120.0 (4)
C15—U1—C4	88.0 (2)	C11—C15—C14	107.7 (6)
C12—U1—C4	137.3 (2)	C11—C15—C20	123.8 (8)
C1—U1—C4	49.4 (2)	C14—C15—C20	127.8 (8)
C3—U1—C4	29.99 (19)	C11—C15—U1	74.6 (4)
F1—U1—C14	128.42 (18)	C14—C15—U1	75.5 (3)
C5—U1—C14	123.0 (2)	C20—C15—U1	123.5 (5)
C11—U1—C14	49.9 (2)	C11—C16—H16A	109.5
C15—U1—C14	30.33 (19)	C11—C16—H16B	109.5
C12—U1—C14	49.38 (19)	H16A—C16—H16B	109.5
C1—U1—C14	143.59 (19)	C11—C16—H16C	109.5
C3—U1—C14	95.79 (18)	H16A—C16—H16C	109.5
C4—U1—C14	95.8 (2)	H16B—C16—H16C	109.5
F1—U1—C2	108.56 (18)	C12—C17—H17A	109.5
C5—U1—C2	49.49 (19)	C12—C17—H17B	109.5
C11—U1—C2	160.6 (2)	H17A—C17—H17B	109.5
C15—U1—C2	132.6 (2)	C12—C17—H17C	109.5
C12—U1—C2	163.9 (2)	H17A—C17—H17C	109.5
C1—U1—C2	30.00 (18)	H17B—C17—H17C	109.5
C3—U1—C2	29.58 (18)	C13—C18—H18A	109.5
C4—U1—C2	49.20 (19)	C13—C18—H18B	109.5
C14—U1—C2	122.10 (19)	H18A—C18—H18B	109.5
F1—U1—C13	112.51 (18)	C13—C18—H18C	109.5
C5—U1—C13	152.4 (2)	H18A—C18—H18C	109.5
C11—U1—C13	49.4 (2)	H18B—C18—H18C	109.5
C15—U1—C13	49.38 (19)	C14—C19—H19A	109.5
C12—U1—C13	29.9 (2)	C14—C19—H19B	109.5
C1—U1—C13	165.14 (19)	H19A—C19—H19B	109.5
C3—U1—C13	117.94 (19)	C14—C19—H19C	109.5
C4—U1—C13	125.0 (2)	H19A—C19—H19C	109.5
C14—U1—C13	29.71 (18)	H19B—C19—H19C	109.5
C2—U1—C13	135.15 (19)	C15—C20—H20A	109.5
F1—U1—Ge1	88.92 (14)	C15—C20—H20B	109.5
C5—U1—Ge1	120.59 (16)	H20A—C20—H20B	109.5
C11—U1—Ge1	119.17 (19)	C15—C20—H20C	109.5
C15—U1—Ge1	133.34 (14)	H20A—C20—H20C	109.5
C12—U1—Ge1	90.33 (16)	H20B—C20—H20C	109.5
C1—U1—Ge1	90.88 (15)	C26—C21—C22	116.9 (5)
C3—U1—Ge1	100.65 (14)	C26—C21—Ge1	123.0 (4)
C4—U1—Ge1	128.17 (14)	C22—C21—Ge1	119.9 (4)
C14—U1—Ge1	108.11 (14)	C23—C22—C21	122.0 (5)
C2—U1—Ge1	79.47 (13)	C23—C22—H22	119.0
C13—U1—Ge1	84.01 (13)	C21—C22—H22	119.0
C21—Ge1—C37	102.4 (3)	C24—C23—C22	120.0 (5)
C21—Ge1—C29	101.7 (2)	C24—C23—C27	120.1 (5)
C37—Ge1—C29	97.9 (3)	C22—C23—C27	119.6 (5)
C21—Ge1—U1	116.65 (17)	C25—C24—C23	118.5 (6)
C37—Ge1—U1	117.2 (2)	C25—C24—H24	120.7
C29—Ge1—U1	117.87 (16)	C23—C24—H24	120.7
C5—C1—C2	108.5 (6)	C26—C25—C24	121.1 (6)
C5—C1—C6	126.6 (6)	C26—C25—C28	119.1 (6)
C2—C1—C6	124.8 (6)	C24—C25—C28	119.8 (6)
C5—C1—U1	73.9 (4)	C25—C26—C21	121.4 (6)
C2—C1—U1	75.8 (3)	C25—C26—H26	119.3
C6—C1—U1	119.6 (4)	C21—C26—H26	119.3
C3—C2—C1	107.9 (6)	F4—C27—F2	108.1 (5)
C3—C2—C7	123.3 (6)	F4—C27—F3	105.9 (5)
C1—C2—C7	127.9 (6)	F2—C27—F3	104.8 (5)
C3—C2—U1	74.5 (3)	F4—C27—C23	113.8 (5)
C1—C2—U1	74.2 (3)	F2—C27—C23	112.2 (5)
C7—C2—U1	125.6 (4)	F3—C27—C23	111.6 (5)
C2—C3—C4	108.5 (6)	F6—C28—F5	108.5 (7)
C2—C3—C8	124.4 (6)	F6—C28—F7	104.0 (7)
C4—C3—C8	126.6 (6)	F5—C28—F7	102.6 (7)
C2—C3—U1	75.9 (3)	F6—C28—C25	113.0 (6)
C4—C3—U1	75.1 (3)	F5—C28—C25	114.5 (6)
C8—C3—U1	122.1 (4)	F7—C28—C25	113.2 (6)
C3—C4—C5	107.1 (6)	C30—C29—C34	117.3 (5)
C3—C4—C9	125.6 (7)	C30—C29—Ge1	124.1 (4)
C5—C4—C9	126.2 (7)	C34—C29—Ge1	118.5 (4)
C3—C4—U1	74.9 (3)	C29—C30—C31	121.2 (5)
C5—C4—U1	73.3 (4)	C29—C30—H30	119.4
C9—C4—U1	126.3 (5)	C31—C30—H30	119.4
C1—C5—C4	108.0 (6)	C32—C31—C30	120.4 (6)
C1—C5—C10	125.1 (7)	C32—C31—C35	119.6 (5)
C4—C5—C10	126.8 (7)	C30—C31—C35	120.0 (5)
C1—C5—U1	76.4 (4)	C31—C32—C33	119.2 (5)
C4—C5—U1	76.2 (4)	C31—C32—H32	120.4
C10—C5—U1	117.0 (5)	C33—C32—H32	120.4
C1—C6—H6A	109.5	C34—C33—C32	120.8 (6)
C1—C6—H6B	109.5	C34—C33—C36	119.2 (6)
H6A—C6—H6B	109.5	C32—C33—C36	120.0 (5)
C1—C6—H6C	109.5	C33—C34—C29	121.2 (6)
H6A—C6—H6C	109.5	C33—C34—H34	119.4
H6B—C6—H6C	109.5	C29—C34—H34	119.4
C2—C7—H7A	109.5	F9—C35—F8	107.1 (6)
C2—C7—H7B	109.5	F9—C35—F10	106.0 (6)
H7A—C7—H7B	109.5	F8—C35—F10	105.2 (6)
C2—C7—H7C	109.5	F9—C35—C31	113.7 (6)
H7A—C7—H7C	109.5	F8—C35—C31	113.0 (5)
H7B—C7—H7C	109.5	F10—C35—C31	111.3 (6)
C3—C8—H8A	109.5	F2A—C36—F1A	115.9 (18)
C3—C8—H8B	109.5	F13—C36—F12	110.8 (8)
H8A—C8—H8B	109.5	F13—C36—F11	103.0 (7)
C3—C8—H8C	109.5	F12—C36—F11	102.0 (7)
H8A—C8—H8C	109.5	F2A—C36—F3A	103.2 (15)
H8B—C8—H8C	109.5	F1A—C36—F3A	98.2 (13)
C4—C9—H9A	109.5	F2A—C36—C33	115.4 (12)
C4—C9—H9B	109.5	F1A—C36—C33	114.2 (12)
H9A—C9—H9B	109.5	F13—C36—C33	114.6 (6)
C4—C9—H9C	109.5	F12—C36—C33	113.1 (6)
H9A—C9—H9C	109.5	F11—C36—C33	112.2 (6)
H9B—C9—H9C	109.5	F3A—C36—C33	107.2 (10)
C5—C10—H10A	109.5	Ge1—C37—H37A	109.5
C5—C10—H10B	109.5	Ge1—C37—H37B	109.5
H10A—C10—H10B	109.5	H37A—C37—H37B	109.5
C5—C10—H10C	109.5	Ge1—C37—H37C	109.5
H10A—C10—H10C	109.5	H37A—C37—H37C	109.5
H10B—C10—H10C	109.5	H37B—C37—H37C	109.5

**Table:**

Contact	Distance (*D*···*A*)	Distance (*D*—H···*A*)
1		
Ge1—H···I1	4.5876 (9)	3.16 (8)
C7—H···F12	3.314 (9)	2.524
C16—H···C30	3.70 (1)	2.79
C20*B*—H···F7	3.44 (1)	2.65
		
2		
C7—H···F3	3.403 (8)	2.652
C7—H···C24	3.536 (9)	2.863
C7···C7	3.36 (1)	
C10—H···F3	3.216 (8)	2.617
C16—H···C19	3.84 (1)	2.87
C18—H···F10	3.446 (8)	2.543
C20—H···C11	3.57 (1)	2.80
C20—H···C12	3.654 (9)	2.752

H···*A* distances involving riding H atoms are rounded to the precision of the *D*···*A* distance.
